# Prevalence and associated factors of Somali teenage pregnancy: a multilevel analysis using the evidence from the demographic and health survey

**DOI:** 10.3389/frph.2026.1831779

**Published:** 2026-05-13

**Authors:** Hamse Adam Abdi, Hamse Arab Ali, Abdulkadir Mohamed Nuh, Abdisalam Hassan Muse

**Affiliations:** 1 University of Hargeisa College of Medicine and Health Sciences, Hargeisa, Somaliland; 2 Amoud University, Borama, Somaliland; 3 HIR Institute for Research and Development, Hargeisa, Somaliland

**Keywords:** adolescents, multilevel analysis, reproductive health, SDHS 2020, Somalia, teenage pregnancy

## Abstract

**Background:**

Teenage pregnancy is a major public health concern that contributes to maternal and neonatal morbidity and mortality, particularly in low–income countries. In Somalia, evidence on the prevalence and determinants of teenage pregnancy is limited to a few studies. This study aimed to assess the prevalence of teenage pregnancy among teenagers aged 15–19 years and the factors associated with it.

**Methods:**

A secondary analysis of the 2020 Somali Demographic and Health Survey (SDHS) was conducted, including 6,608 teenage pregnancies. Descriptive statistics were used to summarise the participant characteristics. Bivariate analyses were used to identify candidate variables (*p* < 0.20) for multivariable analysis. Multilevel mixed-effects logistic regression was used to examine the individual- and community-level factors associated with teenage pregnancy, with results presented as adjusted odds ratios (AORs) and 95% confidence intervals (CIs).

**Results:**

The prevalence of teenage pregnancy was 49.49%. In the full multilevel model, individual-level factors significantly In the full multilevel mixed-effects logistic regression model (Model 3), primary education was associated with higher odds of teenage pregnancy (aOR = 1.27, *p* < 0.05), higher education with lower odds (aOR = 0.46, *p* < 0.01), higher wealth quintiles with reduced odds (aOR = 0.71–0.61, *p* < 0.01 to *p* < 0.001), non-intention to use contraceptives with lower odds (aOR = 0.57, *p* < 0.05), and significant regional variations were observed across Somalia compared to Awdal (aOR = 1.41-3.63, *p* < 0.05), while contraceptive use intention categories showed mixed non-significant effects except non-intention which remained significant (aOR = 0.57, *p* < 0.05) are associated with Teenage Pregnancy. Interventions targeting reproductive health education, contraceptive access, and regional sociocultural differences are essential to reduce teenage pregnancies and achieve related Sustainable Development Goals.

**Conclusion:**

Teenage pregnancy in Somalia is very high (49.49%). It is influenced by education, wealth, contraceptive intentions, and regional differences. Primary education increases risk, while higher education and greater wealth reduce it. Regional disparities are significant, and only the lack of intention to use contraceptives shows a meaningful effect.

## 1 Introduction

The transitional phase from childhood to adulthood is commonly identified as adolescence. According to the World Health Organization (WHO), individuals aged 10–19 years are classified as teenagers (1). Teenage pregnancy refers to pregnancies occurring among females aged 10–19 years (2, 3). This developmental stage is marked by substantial physical, cognitive, and social changes.

Teenagers comprise approximately 20% of the global population, equating to around 1.2 billion individuals (4). Teenage pregnancy is a significant public health concern worldwide. Each year, approximately 21 million girls aged 15–19 years in developing countries become pregnant, with approximately 19 million giving birth. Between 2010 and 2014, approximately 44.5% of pregnancies were unintended and 23% of births were unplanned. Nearly half of these unintended pregnancies result in unsafe abortions, which significantly contribute to maternal mortality (5, 6).

According to estimates by the World Health Organization, approximately 16 million teenage aged 15–19 years and 2 million girls under the age of 15 give birth annually in low- and middle-income countries, with over 90% of these births occurring in these regions (7–9). Sub-Saharan Africa experiences the highest global burden, with more than half of all births attributed to teenage mothers in this region. The teenage birth rate in this region is estimated at 101 per 1,000 girls aged 15–19 years, nearly double the global average (10, 11).

Teenage pregnancy is closely associated with significant maternal and neonatal health challenges. Complications arising from pregnancy and childbirth remain the predominant causes of mortality among females aged 15–19 years (12). Young mothers face an elevated risk of experiencing complications such as eclampsia, postpartum infection, and uterine issues. Their infants are also predisposed to adverse outcomes, including low birth weight, prematurity, and other neonatal complications (13). Young mothers face an elevated risk of experiencing complications such as eclampsia, postpartum infection, and uterine issues. Their infants are also predisposed to adverse outcomes, including low birth weight, prematurity, and other neonatal complications (14, 15).

The Social Ecological Model (SEM) offers a comprehensive framework for analysing the multifaceted and multilevel factors that influence teenage pregnancy, making it particularly suitable for investigating the determinants of teenage pregnancy in Somalia. This model emphasises the interaction between individual and community-level factors that collectively contribute to the risk of teenage pregnancy (16).

Beyond health outcomes, teenage pregnancies have profound social and economic repercussions. It is strongly associated with increased school dropout rates, reduced educational attainment, poverty and limited future employment opportunities. Additionally, it increases vulnerability to risky behaviours and places a burden on child welfare systems. At the community level, high rates of teenage pregnancy negatively affect demographic trends, economic productivity, and overall social development (17–19).

Teenage pregnancy is influenced by a myriad of interrelated risk factors. Research suggests that the incorrect or inconsistent use of contraceptives is often impacted by limited knowledge, misconceptions, and cultural disapproval of premarital sexual activity. Additional contributing factors include early marriage, low educational attainment, poverty, substance use, peer pressure, poor academic performance, lack of access to youth-friendly health services, and restricted decision-making autonomy among teenagers (5, 20–25).

In Somalia, the elevated rate of teenage fertility renders teenage pregnancy a significant public health issue, exerting substantial pressure on the nation's reproductive health services. Teenage pregnancy is a primary contributor to maternal and neonatal morbidity and mortality worldwide. Despite the gravity of this issue, research on teenage pregnancy in Somalia, particularly concerning the underlying factors contributing to early pregnancy among teenage, is limited. Previous studies have identified several determinants of teenage pregnancy, including maternal educational attainment and socioeconomic status; however, evidence specific to the Somali context is scarce. In alignment with Sustainable Development Goals (SDGs) 1, 4, and 5, which emphasise poverty reduction, education, and gender equality, addressing teenage pregnancy is crucial for enhancing teenage health and social outcomes (26).

Empirical studies from various sub-Saharan African nations, such as Ghana, South Africa, and Tanzania, reveal a notable association between poverty and the increased vulnerability of teenage to coerced sexual relationships, often driven by the imperative to satisfy essential financial needs. In a similar vein, child marriage is widely acknowledged as a significant contributor to the high rates of teenage births in countries such as Congo and other regions in Central Africa, where early marriages are common. Further research indicates that girls who enter child marriages often have low levels of educational attainment, come from economically disadvantaged households, and live in rural areas, all of which substantially heighten their risk of early pregnancies. Additionally, social and cultural expectations from families and communities to marry at a young age and demonstrate fertility are crucial factors influencing teenage pregnancy in many sub-Saharan African settings (27).

Despite ongoing reforms under the National Transformation Plan (NTP 2025–2029), which aims to enhance maternal and child health, expand universal health coverage, and improve health information systems, teenage pregnancy remains an under-researched and inadequately addressed public health challenge in Somalia. The country continues to experience high teenage fertility rates within the context of weak health system capacity, limited access to youth-friendly reproductive health services, low skilled birth attendance, and fragmented health information systems, all of which hinder effective prevention and response strategies. Although the NTP acknowledges structural barriers such as inadequate service coverage, poor emergency obstetric care, and persistent socioeconomic inequalities, there is limited disaggregated evidence on how these factors specifically influence teenage pregnancy outcomes in South Africa. Furthermore, existing studies from other sub-Saharan African countries highlight the roles of poverty, early marriage, low education, and sociocultural norms in driving teenage pregnancy. However, these findings are not fully generalisable to Somalia because of differences in conflict context, displacement patterns, and health system functionality. Additionally, most available evidence focuses on individual-level determinants and lacks comprehensive multilevel analyses that incorporate household, community, and health system factors. Therefore, a critical research gap remains in generating nationally representative, context-specific, and analytically robust evidence on the prevalence and multilevel determinants of teenage pregnancy in Somalia to inform targeted interventions and support the effective implementation of the NTP 2025–2029 health priorities (28).

Despite the increasing body of evidence from other sub-Saharan African nations, findings from these regions may not be entirely applicable to Somalia because of variations in sociocultural norms, economic conditions, and marital practices. Furthermore, numerous prior studies have predominantly focused on individual determinants and contextual factors. This deficiency in comprehensive, country-specific evidence constrains policymakers and health programs in their capacity to design targeted interventions that address the underlying causes of teenage pregnancy in Somalia. The methodological gap is evident in the limited utilisation of nationally representative data and multilevel analytical approaches, with most existing studies relying on cross-sectional designs and individual-level analyses that fail to capture the hierarchical determinants of teenage pregnancy in Somalia. Consequently, context-specific and comprehensive research is imperative to enhance our understanding of the prevalence and determinants of teenage pregnancy in Somalia. Addressing this research gap will provide crucial evidence to inform policy development, strengthen teenage reproductive health programs, and support interventions aimed at reducing teenage pregnancy and improving maternal and child health outcomes in the country.

## 2 Methods

### 2.1 Study design and population

This study analysed the Somali Demographic and Health Survey 2020 (SDHS 2020), the first national household survey conducted in Somalia, which collected demographic and health data from 16 regions of the country. The survey provided estimates at the national, regional (18 pre-war areas), and population-type levels (urban, rural, and nomadic) across Somalia's diverse landscapes. The sampling methodology combined cluster sampling with multistage stratified probability sampling, utilising three-stage stratified sampling for urban and rural areas and two-stage sampling for the nomadic regions. Somalia was divided into 55 sampling strata across urban, rural, and nomadic classifications, with Banadir being exclusively urban. Due to security concerns, eight strata were excluded, including three from the Lower Shabelle and Middle Juba regions (29).

### 2.2 Study variables

#### 2.2.1 Outcome variables

This study aimed to examine the status of teenage pregnancy, defined as the proportion of teenagers who are mothers, expect their first child, or have initiated childbearing. This cohort comprised all females aged 15–19 years at the time of the interview (30). Teenage pregnancy, also referred to as teenage pregnancy, was characterised as giving birth before reaching the age of 20 years and was coded as 1 (yes) or 0 (no). This classification was derived from DHS self-reported data, including age at first birth (V212), current pregnancy status (V213), and history of a terminated pregnancy (V228). Any evidence of a birth, ongoing pregnancy, or pregnancy termination during teenage or within five years of the survey was considered a teenage pregnancy. The measure relies on standardised DHS reproductive history data but may be influenced by reporting bias, particularly age misreporting in regions where early marriage is restricted and underreporting of pregnancies in stigmatized environments. Multicollinearity was assessed using the VIF, with all variables exhibiting values below 3 and an average VIF of 1.34, indicating no significant collinearity.

##### 2.2.1.1 Explanatory variables

The independent variables included in the analysis were classified into individual and community levels, where region and place of residence were named community-level factors, and individual-level factors were educational level, listening to radio, watching television, ever used internet, wealth index, knowledge of the ovulatory cycle, contraceptive use, and intention.

### 2.3 Study sample size

**Figure 1:**
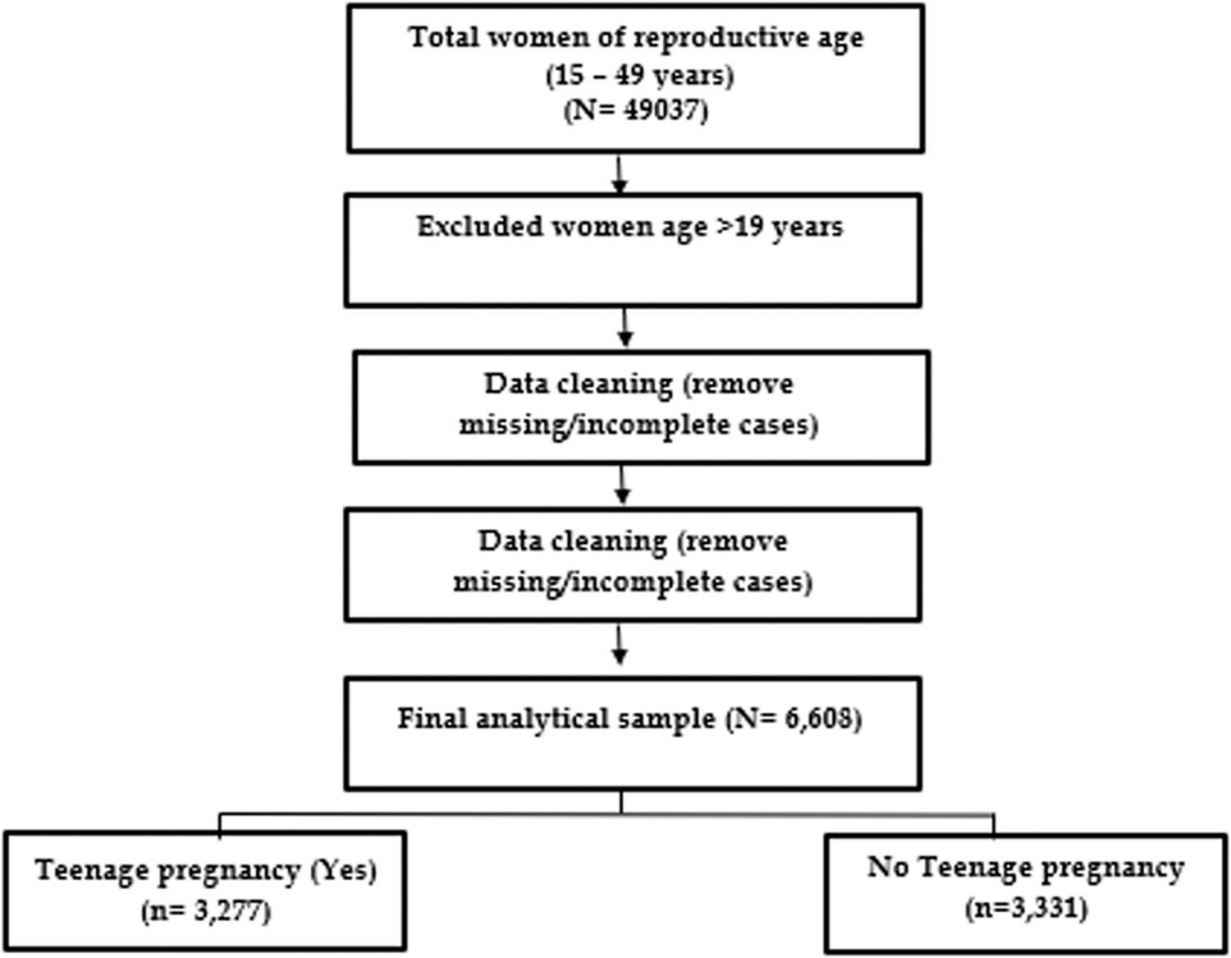
Sampling and exclusion procedures to identify the final sample size in SDHS.2020.

As illustrated in Figure 1, following the data cleaning process, an initial total of 49,037 women of reproductive age were identified from the dataset. In preparation for this study, a dataset comprising 6,608 pregnant teenagers was utilized, with eligible respondents being excluded to ensure the analysis focused strictly on teenagers within the standard definition (10 to 19 years) as per the Somali Demographic and Health Survey (SDHS), deliberately excluding individuals over 19 years of age. The sampling strategy was designed to encompass 16 geographical regions, covering urban, rural, and nomadic areas, serving as stratification layers along with various residential categories.

### 2.4 Data analysis

Data were analyzed using STATA/SE version 16.0 software. The dataset from the most recent DHS was meticulously handled for missing data through a listwise deletion. In Stata, observations with missing values for any of the variables included in the analysis were excluded, ensuring that only complete cases were retained for the final regression analyses. Univariate and bivariate analyses were conducted using cross-tabulations to explore the relationship between each independent variable and teenage pregnancy (coded 0 = no, 1 = yes), with a significance threshold of Variables with a ***p*-value ≤ 0.20** in the chi-square test were considered candidates for inclusion in the multilevel analysis, while statistical significance in the final model was declared at *p* **<** **0.05**. A multicollinearity test was employed to evaluate the correlation between the significance of the variables and the presence of multicollinearity among them. Only those variables that satisfied the statistical criteria (*p* < 0.05) and exhibited acceptable levels of multicollinearity (VIF < 5) were included in the final multilevel analysis. Given the hierarchical structure of the DHS data, where women are nested within households and households are nested within clusters, the assumptions of conventional logistic regression independent observations and equal variance across clusters are violated. Consequently, a multilevel mixed-effects logistic regression was employed to account for clustering at the community level. Four models were fitted: Model 0 (null model) contained only the outcome variable to assess the extent of clustering of teenage pregnancies across clusters. Model I includes only individual-level variables. Model II includes only community-level variables. Model III (full model) incorporated both individual- and community-level variables. The intra-class correlation coefficient (ICC) was calculated to quantify the proportion of variance in teenage pregnancies attributable to differences between clusters. The best-fitting model was identified using the lowest deviance and was supported by the Akaike Information Criterion (AIC) and Bayesian Information Criterion (BIC) values. Adjusted odds ratios (AORs) with 95% confidence intervals (CIs) were used to report the strength and direction of associations, with statistical significance set at *p* < 0.05. Data were weighted to ensure sample representativeness and obtain reliable estimates and standard errors. The prevalence of teenage pregnancy among Somali women was determined using the individual weights of women (v005).

## 3 Results

 Table 1 presents the distribution of women according to their background characteristics and their association with teenage pregnancy. The relationships were assessed using the chi-square (*χ*^2^) test, with statistical significance set at *p* **≤** **0.20**.The results indicate that all examined variables were **statistically significant at the 20% level**. There was a strong regional variation (*χ*^2^ = 163.37, *p* = 0.001), with the highest proportion of teenage pregnancy observed in Banadir (14.40%), followed by Togdheer (9.37%) and Sanaag (9.25%), while Awdal recorded the lowest proportion (3.57%). Educational attainment was also significantly associated with teenage pregnancy (*χ*^2^ = 39.32, *p* = 0.001), with the highest proportion among women with no education (80.16%) and the lowest among those with higher education (1.01%). Similarly, frequency of television viewing showed a statistically significant association (*χ*^2^ = 9.30, *p* = 0.010), as did Internet use (*χ*^2^ = 4.15, *p* = 0.042). Wealth quintile was also significantly associated with teenage pregnancy (*χ*^2^ = 48.65, *p* = 0.001), with variations observed across all wealth categories.

**Table 1:** Distribution of teenage women's characteristics and bivariate association with teenage pregnancy, Somalia DHS 2020.

Variable	Category	Yes n (%)	No n (%)	*χ*^2^ (*p*-value)
Region	Awdal	117 (3.57)	236 (7.08)	163.37 (0.001)
	Woqooyi Galbeed	245 (7.48)	379 (11.38)	
	Togdheer	307 (9.37)	402 (12.07)	
	Sool	233 (7.11)	224 (6.72)	
	Sanaag	303 (9.25)	303 (9.10)	
	Bari	137 (4.18)	170 (5.10)	
	Nugaal	123 (3.75)	119 (3.57)	
	Mudug	170 (5.19)	179 (5.37)	
	Galgaduud	217 (6.62)	173 (5.19)	
	Hiraan	211 (6.44)	182 (5.46)	
	Middle Shabelle	153 (4.67)	98 (2.94)	
	Banadir	472 (14.40)	342 (10.27)	
	Bay	197 (6.01)	143 (4.29)	
	Bakool	129 (3.94)	98 (2.94)	
	Gedo	149 (4.55)	217 (6.51)	
Residence	Urban	1,602 (48.89)	1,597 (47.94)	0.61 (0.737)
	Rural	952 (29.05)	981 (29.45)	
	Nomadic	723 (22.06)	753 (22.61)	
Education	No education	2,627 (80.16)	2,630 (78.96)	39.32 (0.001)
	Primary	484 (14.77)	448 (13.45)	
	Secondary	133 (4.06)	148 (4.44)	
	Higher	33 (1.01)	105 (3.15)	
Frequency of listening to radio	At least once a week	284 (8.67)	267 (8.02)	1.75 (0.417)
	Less than once a week	124 (3.78)	113 (3.39)	
	Not at all	2,869 (87.55)	2,951 (88.59)	
Frequency of watching TV	At least once a week	348 (10.62)	434 (13.03)	9.30 (0.010)
	Less than once a week	117 (3.57)	111 (3.33)	
	Not at all	2,812 (85.81)	2,786 (83.64)	
Owns mobile phone	Yes	2,777 (84.74)	2,804 (84.18)	0.40 (0.528)
	No	500 (15.26)	527 (15.82)	
Ever used internet	Yes	416 (12.69)	480 (14.41)	4.15 (0.042)
	No	2,861 (87.31)	2,851 (85.59)	
Wealth quintile	Lowest	620 (18.92)	611 (18.34)	48.65 (0.001)
	Second	619 (18.89)	583 (17.50)	
	Middle	731 (22.31)	588 (17.65)	
	Fourth	657 (20.05)	678 (20.35)	
	Highest	650 (19.84)	871 (26.15)	
Knowledge of ovulatory cycle	Just before ovulation	1,044 (31.86)	1,055 (31.67)	3.44 (0.486)
	Right after	1,472 (44.92)	1,451 (43.56)	
	Halfway	689 (21.03)	751 (22.55)	
	Other	0 (0.00)	1 (0.03)	
	Don't know	72 (2.20)	73 (2.19)	
Contraceptive use	Modern method	50 (2.17)	40 (1.75)	2.45 (0.484)
	Traditional	1 (0.04)	1 (0.04)	
	Non-user	213 (9.25)	190 (8.31)	
	Does not intend	2,038 (88.53)	2,056 (89.90)	
Marital status	Married	2,854 (87.09)	2,925 (87.81)	0.95 (0.622)
	Divorced	287 (8.76)	270 (8.11)	
	Widowed	136 (4.15)	136 (4.08)	

Table 2 presents a summary of the random effects and model fit statistics for the multilevel logistic regression models evaluating teenage pregnancy among Somali women in the study. The analysis of teenage pregnancy (yes/no) revealed a progressive enhancement in the model fit across the four models. The initial model (Model 0) exhibited an ICC of 0.0234, an AIC of 9148.82, and a BIC of 9,162.41, indicating initial clustering effects. Upon the inclusion of individual-level variables, Model 1 demonstrated reduced clustering with an ICC of 0.0202 and an improved fit, as evidenced by lower AIC (6,337.60) and BIC (6,485.52) values. Model 2, which incorporated community-level variables, further decreased the ICC to 0.0046, with AIC (9,021.99) and BIC (9,151.11) values lower than those of Model 0 but higher than those of Model 1. The comprehensive model (Model 3), which integrated both individual- and community-level variables, exhibited the lowest ICC (0.001) and the smallest AIC (6,236.52) and BIC (6,487.33), indicating an optimal overall model fit. Consequently, Model 3 was selected as the final and most suitable model for interpreting the factors associated with teenage pregnancies.

**Table 2:** Model comparison and random effect analysis result of teenage pregnancy in Somali women.

Parameter	Model 0	Model 1	Model 2	Model 3
Intercept (Constant)	0.97	1.91^*^	0.44^***^	1.01
V001 (Cluster) Variance	0.0789	0.0680	0.0151	0.0007
ICC	0.0234	0.0202	0.0046	0.001
Model Fit				
Observations (N)	6,608	4,588	6,608	4,588
Groups (clusters)	308	306	308	306
Log Likelihood	−4,572.41	−3,145.80	−4,491.99	−3,079.26
AIC	9,148.82	6,337.60	9,021.99	6,236.52
BIC	9,162.41	6,485.52	9,151.11	6,487.33

*p < 0.05 (statistically significant).

***p < 0.001 (highly significant).

The intraclass correlation coefficient (ICC) was used to evaluate the proportion of total variation in teenage pregnancies attributable to inter-cluster differences. In the baseline model (Model 0), the ICC was 0.0234, indicating that approximately 2.34% of the variation in teenage pregnancy was due to differences between clusters. Upon incorporating individual-level factors in Model 1, the ICC slightly decreased to 0.0202, suggesting a minor reduction in the unexplained cluster-level variation. With the inclusion of community-level variables in Model 2, the ICC further declined to 0.0046, indicating that the majority of the between-cluster variation was accounted for by the included covariates. In the fully adjusted model (Model 3), the ICC approached (0.001), demonstrating that virtually no residual clustering remained after controlling for both individual and community-level determinants of the outcome.

Table 3: In the comprehensive multilevel mixed-effects logistic regression model (Model 3), several factors at both the individual and community levels were significantly correlated with teen pregnancy. Teenage with primary education exhibited higher odds of experiencing teenage pregnancy compared to those with no education (aOR = 1.27, *p* < 0.05), whereas those with higher education demonstrated significantly lower odds (aOR = 0.46, *p* < 0.01). Household wealth also emerged as a significant factor, with teenagers from the fourth wealth quintile (aOR = 0.71, *p* < 0.01) and the highest wealth quintile (aOR = 0.61, *p* < 0.001) showing reduced odds of teenage pregnancy compared to those from the lowest wealth group. Furthermore, teenage who did not intend to use contraceptives had lower odds of teenage pregnancy than those currently using modern contraceptives (aOR = 0.57, *p* < 0.05). At the community level, significant regional disparities were identified, with teenage residing in Togdheer (aOR = 1.41), Sool (aOR = 2.02), Sanaag (aOR = 1.94), Nugaal (OR = 2.17), Mudug (aOR = 1.65), Galgaduud (aOR = 2.21), Hiraan (aOR = 2.29), Middle Shabelle (aOR = 3.12), Banadir (aOR = 3.33), Bay (aOR = 3.63), Bakool (aOR = 2.59), and Gedo (aOR = 1.92) exhibiting significantly higher odds of teenage pregnancy compared to those in the Awdal region. In Model 3, compared to teenage currently using modern contraceptives, those employing traditional methods (aOR = 1.06) showed no significant difference, those intending to use later (aOR = 0.77) had lower but non-significant odds, and those who did not intend to use contraceptives had significantly lower odds of teenage pregnancy (aOR = 0.57), indicating an approximately 43% reduction in odds.

**Table 3:** Multi-Level logistic regression models for predicting teenage pregnancy among Somali women.

Variable	Category	Model 0 (Null) OR	Model 1 (Individual) OR	Model 2 (Community) OR	Model 3 (Full) OR
Individual-Level					
Education (V106)	No education (ref)		1.00		1.00
	Primary		1.18		1.27^*^
	Secondary		1.04		1.09
	Higher		0.43^**^		0.46^**^
Media: Read newspaper (V158)	At least once a week (ref)		1.00		1.00
	Less than once a week		0.93		0.93
	Not at all		0.87		0.98
Media: Listen radio (V159)	At least once a week (ref)		1.00		1.00
	Less than once a week		1.41		1.37
	Not at all		1.07		0.98
Media: Watch TV (V169A)	Yes (ref)		1.00		1.00
	No		0.92		1.02
Mobile phone use (V171)	Yes (ref)		1.00		1.00
	No		1.03		1.21
Wealth index (V190)	Lowest (ref)		1.00		1.00
	Second		0.96		0.83
	Middle		1.09		0.88
	Fourth		0.86		0.71^**^
	Highest		0.70^**^		0.61^***^
Knowledge of ovulatory cycle (V217)	At time of period (ref)		1.00		1.00
	Right after period ends		0.95		0.93
	Half way between periods		0.88		0.86
	Don't know		0.85		0.79
Contraceptive use intention (V364)	Currently using modern (ref)		1.00		1.00
	Using traditional method		0.73		1.06
	Non-user, intends to use later		0.74		0.77
	Does not intend to use		0.62^*^		0.57^*^
Marital status (V501)	Married (ref)		1.00		1.00
	Divorced		1.19		1.23
	Widowed		0.88		0.91
Community-Level					
Region (V101)	Awdal (ref)			1.00	1.00
	Woqooyi Galbeed			1.32	1.28
	Togdheer			1.56^**^	1.41^*^
	Sool			2.07^***^	2.02^***^
	Sanaag			2.07^***^	1.94^***^
	Bari			1.64^**^	1.38
	Nugaal			2.13^***^	2.17^***^
	Mudug			1.95^***^	1.65^*^
	Galgaduud			2.62^***^	2.21^***^
	Hiraan			2.35^***^	2.29^***^
	Middle Shabelle			3.19^***^	3.12^***^
	Banadir			3.11^***^	3.33^***^
	Bay			3.84^***^	3.63^***^
	Bakool			2.86^***^	2.59^***^
	Gedo			2.72^***^	1.92^**^
	Lower Juba			1.45^*^	1.01
Residence (V102)	Urban (ref)			1.00	1.00
	Rural			1.08	1.07
	Nomadic			1.14	1.00

*p < 0.05 (statistically significant).

**p < 0.01 (more significant).

***p < 0.001 (highly significant).

## 4 Discussion

**Figure 2:**
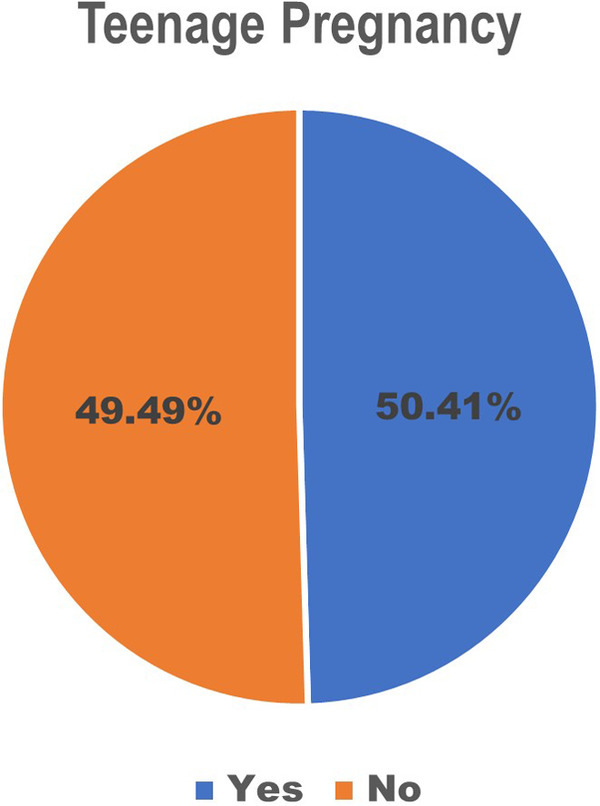
Prevalence of teenage pregnancy.

The findings of this study revealed that the prevalence of teenage pregnancies was 49.49%, as depicted in Figure 2. This rate surpasses those reported in studies conducted in Ethiopia (23.59%), Cameroon (8.7%), Kenya (23.3%), Nigeria (22.9%), and South Africa (19.2%) (13, 31–34). This discrepancy may be attributed to differences in the study populations, as our study included all forms of pregnancy, including terminated pregnancies, such as teenage pregnancies. In contrast, some previous studies may have employed more restrictive definitions of frailty. Furthermore, varying socioeconomic conditions and cultural norms in African countries, such as Somalia, may contribute to these differences (35).

This study identified significant regional disparities in the teenage pregnancy rates. Teenage residing in Togdheer, Sool, Sanaag, Nugaal, Mudug, Galgaduud, Hiraan, Middle Shabelle, Banadir, Bay, Bakool, and Gedo exhibited higher odds of experiencing teenage pregnancy than their counterparts in Awdal, with odds ratios ranging from approximately 1.4 to 3.6. The most pronounced odds were observed in Bay, Banadir, and Middle Shabelle, whereas Togdheer demonstrated the smallest increase among regions with elevated odds. In comparison to a study conducted in Ethiopia, notable regional differences in teenage pregnancies were also evident. Teenage in Amhara were less likely to initiate childbearing, designating it as a protective region, whereas those in Oromiya, Somali, and SNNPR were more prone to commence childbearing, identifying these regions as high-risk areas for teenage pregnancies (36). Potential explanations for this phenomenon include cultural variations in marriage age norms, disparities in economic conditions, educational attainment, access to media, and healthcare infrastructure (37–39).

Teenagers utilising modern contraceptives and those employing traditional methods exhibited comparable probabilities of experiencing teenage pregnancy, approximately 1.1 times. Conversely, individuals intending to use contraceptives at a later stage demonstrated reduced odds (approximately 0.8), while those with no intention to use contraceptives exhibited even lower odds (approximately 0.6). This suggests that the absence of current contraceptive use or delayed utilisation elevates the risk of teenage pregnancies (23). This phenomenon may be attributed to the awareness of modern contraceptive methods among teenage girls after conception. Alternatively, pregnancy may occur despite awareness of family planning due to societal pressures or a desire for motherhood, which external incentives to delay pregnancy do not mitigate. Research conducted in sub-Saharan Africa has demonstrated that increased awareness of modern contraceptive methods, particularly among teenage, does not necessarily correlate with higher contraceptive use rates (40–42). This may be due to cultural barriers, limited access to healthcare facilities or youth-friendly services, teenagers’ restricted autonomy and decision-making power, and social norms that discourage contraceptive use (43).

Education was significantly correlated with the incidence of teenage pregnancy. Teenage with only primary education exhibit approximately 1.3 times greater odds of experiencing teenage pregnancy compared to their counterparts with no formal education. Conversely, those with higher educational attainment demonstrated approximately 0.5 times lower odds. This indicates that elevated educational levels are associated with a reduced risk of teenage pregnancies. These findings align with research conducted in Ghana, which revealed that female teenage who have never attended school are more likely to marry during their teenage years than those with some educational background. Similarly, studies in Brazil have shown that daughters from low-income families frequently do not complete primary education, thereby increasing their risk of teenage pregnancy (44–46).

Household wealth was significantly correlated with the incidence of teenage pregnancy. Teenage from the fourth wealth quintile exhibited approximately 0.7 times lower odds of experiencing teenage pregnancy compared to those in the lowest wealth group, while individuals from the highest wealth quintile demonstrated approximately 0.6 times lower odds. These findings are supported by other studies conducted in Nigeria, Ethiopia, and The Gambia. A systematic review aimed at evaluating the risk factors associated with teenage pregnancy in Sub-Saharan African countries revealed that teenage from lower socioeconomic backgrounds are particularly susceptible to early marriage and pregnancy, as parents often seek to ensure their daughters’ financial security (20, 47, 48).

## 5 Conclusion

The prevalence of teenage pregnancy in Somalia is notably high at 49.49%, surpassing the rates observed in several other African countries. This study aligns with the United Nations’ Sustainable Development Goals 1, 4, and 5. Various factors were significantly associated with teenage pregnancy at both the individual and community levels. Teenage with primary education exhibited higher odds of experiencing teenage pregnancy than those with no education, whereas those with higher education demonstrated lower odds. An increase in household wealth was correlated with a decreased likelihood of teenage pregnancy. Teenagers who did not intend to use contraceptives also had lower odds of teenage pregnancy than those currently utilising modern contraceptives. At the community level, significant regional disparities were evident across Somalia, with teenage residing in several regions displaying considerably higher odds of teenage pregnancy than those in Awdal. Overall, the categories of contraceptive use intention exhibited mixed effects, with most differences not achieving statistical significance, except for those with no intention to use contraceptives, which influenced teenage pregnancy among Somali women in the study.

The study's limitations include the use of a cross-sectional survey design, which provides a snapshot through one-time data collection on teenage pregnancies. This approach, coupled with reliance on self-reported data, may introduce recall bias, thereby limiting the ability to infer causal relationships. To comprehensively elucidate this complex relationship and understand the underlying mechanisms, further longitudinal studies are warranted. The analysis was restricted to teenage aged 15–19 years, which may have resulted in truncation bias. Consequently, some participants may not have completed their teenage years at the time of the survey, potentially leading to an underestimation of the teenage pregnancy rates. Additionally, potential confounders and unobserved variables, such as cultural norms, peer influence, partner characteristics, access to reproductive health services, sexual behaviour, family communication, and media exposure, were not accounted for in the analysis and may have influenced the outcomes related to teenage pregnancies.

## Data Availability

The datasets presented in this study can be found in online repositories. The names of the repository/repositories and accession number(s) can be found in the article/Supplementary Material.
